# A systematic review of post-release programs for women exiting prison with substance-use disorders: assessing current programs and weighing the evidence

**DOI:** 10.1186/s40352-021-00162-6

**Published:** 2022-01-03

**Authors:** Layla Edwards, Sacha Kendall Jamieson, Julia Bowman, Sungwon Chang, Josie Newton, Elizabeth Sullivan

**Affiliations:** 1 School of Public Health, Faculty of Health, University of Technology Sydney, Ultimo, NSW 2007 Australia; 2 Sydney School of Education and Social Work, Faculty of Arts and Social Sciences, The University of Sydney, Sydney, NSW 2006 Australia; 3 Research Operations Manager, Research Unit, Justice Health and Forensic Mental Health Network, Malabar, NSW 2036 Australia; 4 Faculty of Health, University of Technology Sydney, Ultimo, NSW 2007 Australia; 5 School of Public Health, Australian Centre for Public and Population Health Research, University of Technology Sydney, Ultimo, NSW 2007 Australia; 6 Centre for Improving Palliative, Aged and Chronic Care through Clinical Research and Translation (IMPACCT), Faculty of Health, University of Technology Sydney, Ultimo, NSW 2007 Australia; 7 College of Health, Medicine and Wellbeing, University of Newcastle, Callaghan, NSW 2308 Australia; 8 Acting Deputy Vice Chancellor Research, University of Newcastle, Callaghan, NSW 2308 Australia; 9 Custodial Health Justice Health and Forensic Mental Health Network, Malabar, NSW 2036 Australia

**Keywords:** Women, Prisoners, Post-release, Transitional, Intervention, Re-entry program, Program evaluation, Substance-related disorders, Recidivism, Systematic review

## Abstract

**Background:**

The rising rates of women in prison is a serious public health issue. Unlike men, women in prison are characterised by significant histories of trauma, poor mental health, and high rates of substance use disorders (SUDs). Recidivism rates of women have also increased exponentially in the last decade, with substance related offences being the most imprisoned offence worldwide. There is a lack of evidence of the effectiveness of post-release programs for women. The aim of this systematic review is to synthesise and evaluate the evidence on post-release programs for women exiting prison with SUDs.

**Methods:**

We searched eight scientific databases for empirical original research published in English with no date limitation. Studies with an objective to reduce recidivism for adult women (⩾18 years) with a SUD were included. Study quality was assessed using the revised Cochrane Risk of Bias tool for randomized trials (RoB2) and the Risk of Bias in Non-randomized Studies - of Interventions (ROBINS-I) tools.

**Results:**

Of the 1493 articles, twelve (*n* = 3799 women) met the inclusion criteria. Recidivism was significantly reduced in five (42%) programs and substance-use was significantly reduced in one (8.3%) program. Common attributes among programs that reduced recidivism were: transitional, gender-responsive programs; provision of individualised support; providing substance-related therapy, mental health and trauma treatment services. Methodological and reporting biases were common, which impacted our ability to synthesize results further. Recidivism was inconsistently measured across studies further impacting the ability to compare results across studies.

**Conclusions:**

Recidivism is a problematic measure of program efficacy because it is inconsistently measured and deficit-focused, unrecognising of women’s gains in the post-release period despite lack of tailored programs and significant health and social disadvantages. The current evidence suggests that women benefit from continuity of care from prison to the community, which incorporated gender-responsive programming and individualised case management that targeted co-morbid mental health and SUDs. Future program design should incorporate these attributes of successful programs identified in this review to better address the unique challenges that women with SUDs face when they transition back into the community.

**Supplementary Information:**

The online version contains supplementary material available at 10.1186/s40352-021-00162-6.

## Introduction

Incarcerated women are one of the most vulnerable groups in society who, upon entry into prison exhibit a range of complex and inter-related health and social issues (Dumont, Brockmann, Dickman, Alexander, & Rich, 2012; J. E. Johnson & Zlotnick, 2008; Kinner & Young, 2018; Pelissier, Motivans, & Rounds-Bryant, 2005). Although the proportion of incarcerated women globally is much lower than the proportion of incarcerated men (6.9% compared to 93.1%, respectively) (Walmsley, 2017), the number of women imprisoned since 2000 continues to increase globally at a rate that is double the rate for the imprisonment of men (Australian Bureau of Statistics, 2018b; J. E. Johnson & Zlotnick, 2008; B. E. Salem et al., 2013; Walmsley, 2017). There are considerable variations between countries, for example the latest Australian figures show that around 8% (*n* = 3587) of the prison population is women (Australian Bureau of Statistics, 2018a) and in the United Kingdom this figure was 5% (*n* = 7745) (Women in Prison, 2017). The United States has the highest total number of women in prison (*n* = 211,870, representing 8.7%) in any one country, as well as the highest prison population rate for women (about 65.7 per 100,000 of the national population) (World Prison Brief, 2018). Comparatively, African countries have a much lower total prison population proportion at 3.4% (or 3.2 per 100,000 of the national population) (Walmsley, 2017).

### Characteristics of women in prison

Much of this rise is associated with increases in the arrest, prosecution, and incarceration for substance-related offenses (alcohol and other drugs) (Ray, Grommon, Buchanan, Brown, & Watson, 2017). Unlike men, women are typically imprisoned for non-violent offences; with substance-related offences being the most imprisoned offence worldwide (Australian Bureau of Statistics, 2017a; Begun, Rose, & LeBel, 2011; Rushforth & Willis, 2003; World Health Organisation, 2009). The correlation between substance-use and criminal offending has been well researched (Begun et al., 2011; Fearn et al., 2016; H. Johnson, 2006; Moore, Hacker, Oberleitner, & McKee, 2020) and the evidence shows women to have disproportionately higher rates of substance-use disorders (SUDs) compared to men in prison and compared to women in the general community (Begun et al., 2011). A systematic review across ten countries found upon reception to prison the estimated pooled prevalence of alcohol use disorders for women in prison was 20% (95% CI = 16–24) compared to 26% (95% CI = 23–30) for men. The estimated pooled prevalence of drug use disorders was 51% (95% CI = 43–58) for women compared to 30% (95% CI = 22–38) for men (Fazel, Yoon, & Hayes, 2017). Another study reviewed trends in substance-use by gender among people in jail over an 18 year period (1998–2016) (Bello, Hearing, Salas, Weinstock, & Linhorst, 2020). Significant differences in substance-use trends was noted: Heroin (36.4% women vs. 22.0% men *p* < 0.0001) and stimulants (38.0% women vs. 19.6% men, p < 0.0001) were more strongly preferred by women than men while alcohol (49.0% men vs. 29.1% women, p < 0.0001) and marijuana (48.7% men vs. 33.6% women, *p* < 0.0001) were more strongly preferred by men. There was a low overall prevalence for preference of prescription drugs (8.0%), however twice as many women strongly preferred this category compared to men (12.9% women vs. 6.2% men, p < 0.0001) (Bello et al., 2020). Other research has shown that women typically begin SUD treatment with more complex and significant physical, emotional and behavioural needs compared to men (Back et al., 2011; NIDA., 2021). Despite this, women are more likely than men to face multiple barriers affecting access and entry to SUD treatment (Tuchman, 2010).

Along with SUDs, women in prison are characterised by extensive histories of trauma and poor mental health (MH) (Covington, 2001; J. E. Johnson & Zlotnick, 2008; B. E. Salem et al., 2013; Schonbrun, Johnson, Anderson, Caviness, & Stein, 2017; Wetton & Sprackett, 2007; World Health Organisation, 2009). The prevalence of emotional, physical, and sexual abuse is reported between 77% and 90% of women in prison respectively (Australian Institute of Family Studies, 2012; Messina & Grella, 2006). A recent review summarised the literature on sexual abuse and mental illness prevalence among samples of incarcerated women (Karlsson & Zielinski, 2018). Best estimates for sexual abuse were: 50–66% for child sexual abuse, 28–68% for adult sexual abuse, and 56–82% for a lifetime of sexual assault (Karlsson & Zielinski, 2018). The review highlighted that incarcerated women have significantly greater exposure to sexual victimization compared to national standards, incarcerated men and women in community (Karlsson & Zielinski, 2018).

Experiences of trauma predispose women for adverse MH conditions such as post-traumatic stress disorder, depression, anxiety and suicide (Karlsson & Zielinski, 2018; World Health Organisation, 2009). Women who experienced trauma as a child have a 40% increase in odds of developing a MH condition in adulthood (Messina & Grella, 2006). A meta-analysis of the effect of adverse childhood experiences on health describes the findings of 37 studies and presents the pooled risk of various health conditions (Hughes et al., 2017). The risk of adverse MH conditions, such as anxiety, depression, and schizophrenia, was found to be about four times higher, as compared to people who experienced less than four adverse childhood experiences (anxiety OR 3.70; depression OR 4.40, schizophrenia OR 3.60). In addition, people with four or more adverse childhood experiences were at higher risk of SUDs with problematic alcohol use nearly six times higher (OR 5.84) and problematic drug use over ten times as high (OR 10.22) (Hughes et al., 2017). Substance dependency among women in prison is significantly higher among women who have experienced childhood abuse and MH problems (H. Johnson, 2006).

**Fig. 1:**
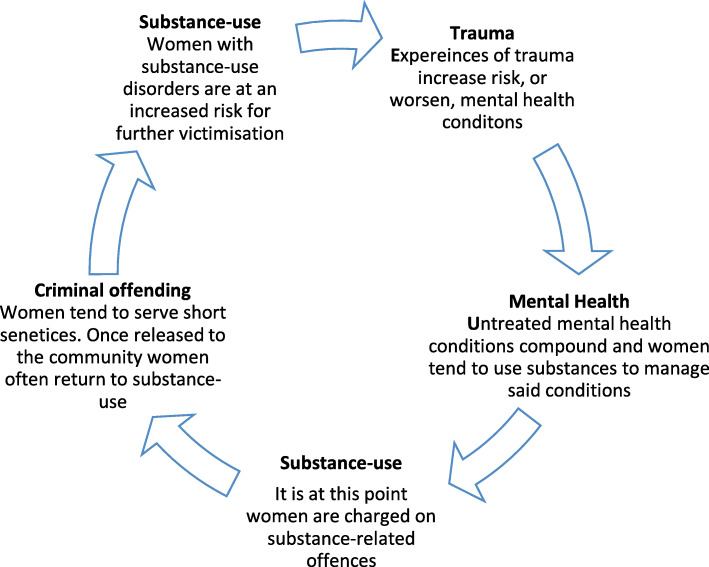
Pathways to women’s criminal offending

Women are more likely than men to start using substances as a means to alleviate the pain of trauma and to manage existing MH conditions (Langan & Pelissier, 2001; Stalans, 2009). Trauma, MH and substance-use are therefore inter-related factors that can result in cumulative and compounding MH issues, addiction, and contact with the criminal justice system (see Fig. 1) (Alleyne, 2008; Australian Bureau of Statistics, 2017b; Covington, 2001; Karlsson & Zielinski, 2018; B. E. Salem et al., 2013).

### Exiting prison

Compared to men, women generally serve short sentences which is a reflection of the minor, non-violent crimes they have been sentenced for (Baldry, 2010; Balyakina et al., 2014; van den Bergh, Gatherer, & Møller, 2009). When women are released into the community they face many disadvantages including poor continuity of care, inadequate social support, parenting stress, homelessness and poverty, and reduced employment opportunities (Baldry, 2010; Begun, Early, & Hodge, 2016; B. E. Salem et al., 2013). A notable difference between men and women in prison is that half of incarcerated women (54%) are mothers to dependent children (age < 16) and were the primary carer of one or more children before incarceration (compared to only 36% for men) (Australian Institute of Health and Welfare, 2019; Kilroy, 2016). Maternal stress, coupled with the many disadvantages cited, are often barriers to accessing immediate and affordable healthcare and drug and alcohol treatment services. As a result, women with SUDs who are recently released from prison are at a high risk of experiencing an adverse MH episode, illness and death compared to the general population. The risk of death is especially high in the first month after release, and the causes of death are usually preventable, including suicide, injury, and overdose (Sullivan et al., 2019).

Post-release (also known as re-entry, reintegration, and resettlement) programs are interventions that are delivered in the community. Transitional programs are interventions that start pre-release (in custody) and support people during the transition from prison to community (Baldry, 2010; Borzycki, 2005). Post-release and transitional programs are often evaluated based on a measurement of recidivism. Recidivism is used to measure the proportion of people who go on to reoffend during a pre-defined post-release period (Bartels & Gaffney, 2011; Sullivan et al., 2019; Urban Institute, n.d.; Yukhnenko, Sridhar, & Fazel, 2019). A systematic review of recidivism rates, two years post-release for both men and women across 11 countries found re-arrest rates were between 26% and 60% and reconviction rates ranged from 20% to 63% (Yukhnenko et al., 2019). These recidivism rates suggest that many people with a history of incarceration either do not access, or do not benefit from services and programs during their time in prison, or do not have adequate support or change in social circumstances in the community to prevent reoffending-arrest (Baldry, McDonnell, Maplestone, & Peeters, 2006).

Despite the growth of the women’s prison population, and their profoundly different criminogenic profile compared to incarcerated men, the majority of prison programs available have been designed for men and extended to women with little alteration (Armstrong, Chartrand, & Baldry, 2005; Bartels & Gaffney, 2011; Langan & Pelissier, 2001; Lawlor, Nicholls, & Sanfilippo, 2008; Suter, Byrne, Byrne, Howells, & Day, 2002). Emerging evidence indicates that community based programs that are gender-responsive and address criminogenic needs can improve the transition process and minimise recidivism rates post-release (Begun et al., 2016; Borzycki, 2005; Borzycki & Baldry, 2003; Carlton & Segrave, 2016). Gender-responsiveness (or gender-informed) refers to programming that explicitly considers the needs that are particularly salient to women. Gender-responsive approaches are trauma-informed and consider the gendered context (or “pathways”) of criminal offending (Covington & Bloom, 2006; Gobeil, Blanchette, & Stewart, 2016). A meta-analytic review of correctional interventions for women in prison examined whether programs, either gender-informed or gender-neutral, were effective in reducing recidivism (Gobeil et al., 2016). The results demonstrated that participation was associated with 22% to 35% greater odds of community success and gender-responsive interventions were significantly more likely to be associated with reductions in recidivism (Gobeil et al., 2016).

Given the proportion of women in prison with SUDs and correlation to reoffending and risk of death post-release, more research is needed to understand the effectiveness of programs for this population. To-date, there has been no systematic review of the evidence about what is available and “what works” in regard to post-release programs for women with SUDs. The aim of this research is to critically review the available evidence of the effectiveness of community based (post-release and transitional) programs offered to women with SUDs to inform program development to decrease reoffending. Further, as the link between criminal offending and substance-use is well established, we also aim to review the effectiveness of interventions to reduce substance-use outcomes post-release and whether this impacts recidivism. This review addresses the following research questions (RQ):

- RQ1: Are post-release and/or transitional programs effective in reducing recidivism and/or substance-use for women with SUDs post-release?
- RQ2: Do those that report a reduction in substance-use also report a reduction in recidivism?
- RQ3: What program characteristics are common among programs which report improved recidivism and substance-use outcomes post-release?

## Methods

This systematic review followed the Preferred Reporting Items for Systematic Reviews and Meta-Analyses (PRISMA) guidelines (Moher, Liberati, Tetzlaff, Altman, & the PRISMA Group, 2009). The systematic review was registered in the PROSPERO database CRD42020162036. The databases PubMed and CINAHL (including MESH terms), Cochrane, EMBASE (including EMTREE terms), Scopus, PsycInfo, ProQuest and SOCIndex were originally searched in September 2019, with no date limitation. The search strategy was split into six core concepts using a combination of words related to “*Post-release*”, “*Prison*”, “*Women*”, and “*Interventions*”. The electronic database searches were supplemented with manual searches of the reference lists from relative articles. Due to the limited number of publications found an updated search was conducted in February 2020 following the method by Bramer and Bain (2017), adding search terms related to “*Substance use*” and “*Recidivism*” (see Additional File 1).

### Eligibility criteria

Studies included were primary reports of effectiveness trials (i.e., studies of an intervention with a comparator) with an objective to reduce recidivism for adult women (⩾18 years) with a known SUD. The program had to be either a post-release or transitional intervention, published in English in a peer-reviewed journal. In this review substance-use included individuals using occasional drugs or alcohol, those who were dependent, or those who had other drug and alcohol related problems prior to their current offence. Studies that included both men and women were included if the results relating to women could be isolated. Due to the limited published studies of women in prison (Baldry, 2010; Borzycki & Baldry, 2003; Segrave & Carlton, 2011) there were no limitations by study design or intervention type to ensure identification of all successful programs. Interventions that were pre-release only (only delivered whilst incarcerated), did not focus on women, were mix gendered and did not report gendered data separately, were excluded. Systematic reviews, meta-analyses, protocol papers and studies that did not evaluate a program were also excluded.

### Data extraction and quality assessment

Search results were imported into Endnote X7 software, duplicates removed, and results exported into Covidence online software. Two investigators independently applied eligibility criteria to titles and abstracts and discrepancies identified through the platform were discussed in a face-to-face meeting. Studies that were included were progressed to full-text review where the investigators systematically went through individual articles thoroughly to check eligibility and documented reasons for exclusion. Discrepancies were resolved through face-to-face discussion and a third reviewer was approached when needed. The lead reviewer extracted data according to the template for intervention description and replication (TIDieR) checklist and guide (Hoffmann, Glasziou, & Boutron, 2014) into a Microsoft Excel spreadsheet.

Finally, the investigators independently evaluated the risk of bias of studies using the revised Cochrane Risk of Bias tool for randomized trials (RoB2) (Sterne JAC et al., 2019) and the Risk of Bias in Non-randomized Studies - of Interventions (ROBINS-I) tool (Sterne JAC et al., 2016) (See supplementary files 1–2 for full version assessment tools). Each study was scored one point for each criterion that was fully met, half a point (0.5) if a criterion was ‘somewhat’ met, and zero for criteria that were either not met (‘no’) or not applicable. Each paper’s score was estimated by summing the criteria scores and dividing the total by the number of applicable fields (excluding those criteria which did not apply) and multiplying by 100. Scores < 0.50 were characterised as ‘low/moderate quality’ and > 0.50 as ‘fair quality’. After the investigators individually assessed studies, they resolved discrepancies through discussion. It should be noted that due to the type of intervention being assessed it was not possible to blind participants, staff, or outcome assessors to participant allocation. We therefore did not score this against the studies performance or detection bias (RoB2 criteria numbers 2.1, 2.2, 4.3; ROBINS-I criteria numbers 6.2).

### Data synthesis

Tables and text were generated to report study and program characteristics and outcomes. An intervention matrix was created, and descriptive numerical analyses were performed using Microsoft Excel.

## Results

**Fig. 2:**
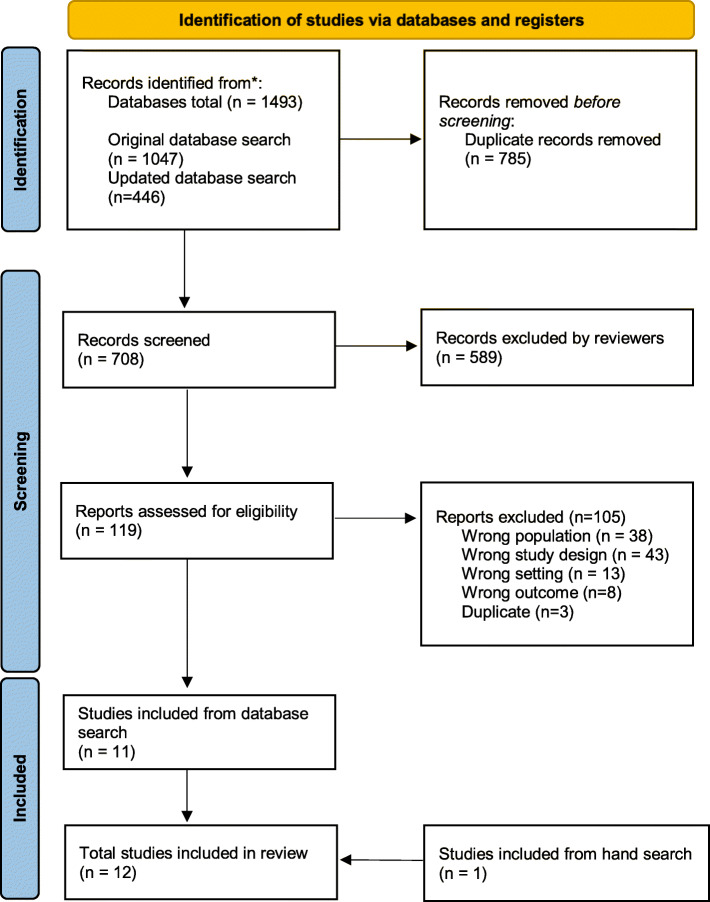
PRISMA diagram

The original database search included 1047 citations and the updated search included in 446 citations, resulting in 1493 citations. After the removal of 785 duplicates and 589 articles through title and abstract screening, we reviewed 119 full text articles, of which 105 were excluded as they did not fit the selection criteria. Eleven articles met the criteria with one additional article included following hand-searching, resulting in a total of 12 articles for review (see Fig. 2).

The 12 studies were conducted between 2005 and 2018 with 11 studies from the United States (Chan et al., 2005; Covington, Burke, Keaton, & Norcott, 2008; Grella & Rodriguez, 2011; Guydish et al., 2011; J. E. Johnson, Friedmann, Green, Harrington, & Taxman, 2011; Messina, Burdon, & Prendergast, 2006; Miller, Miller, & Barnes, 2016; Needels, James-Burdumy, & Burghardt, 2005; Nyamathi et al., 2018; Schram & Morash, 2002; Scott, Dennis, & Lurigio, 2017) and one from Canada (Farrell-Macdonald, Macswain, Cheverie, Tiesmaki, & Fischer, 2014) (see Table 1 and Additional file 2). Most studies were either RCTs (*n* = 5) (Guydish et al., 2011; J. E. Johnson et al., 2011; Needels et al., 2005; Nyamathi et al., 2018; Scott et al., 2017) or quasi-experimental studies (*n* = 4) (Chan et al., 2005; Messina et al., 2006; Miller et al., 2016; Schram & Morash, 2002). There was a total of 4865 participants in the 12 studies with women making up 78% of participants and ranging in mean age from 30.1 to 39.1 years (excluding one study who did not report mean age (Schram & Morash, 2002)).

**Table 1:** Included study characteristics

Characteristics	N (%)	Reference
Inclusion period, years	2002–2018	(Chan et al., 2005; Covington et al., 2008; Farrell-Macdonald et al., 2014; Grella & Rodriguez, 2011; Guydish et al., 2011; J. E. Johnson et al., 2011; Messina et al., 2006; Miller et al., 2016; Needels et al., 2005; Nyamathi et al., 2018; Schram & Morash, 2002; Scott et al., 2017)
Country		
United States	11 (92%)	(Chan et al., 2005; Covington et al., 2008; Grella & Rodriguez, 2011; Guydish et al., 2011; J. E. Johnson et al., 2011; Messina et al., 2006; Miller et al., 2016; Needels et al., 2005; Nyamathi et al., 2018; Schram & Morash, 2002; Scott et al., 2017)
Canada	1 (8%)	(Farrell-Macdonald et al., 2014)
Design		
Randomised control trial	5 (42%)	(Guydish et al., 2011; J. E. Johnson et al., 2011; Needels et al., 2005; Nyamathi et al., 2018; Scott et al., 2017)
Quasi-experimental	4 (33%)	(Chan et al., 2005; Messina et al., 2006; Miller et al., 2016; Schram & Morash, 2002)
One-group pre/post-test design	1 (8%)	(Covington et al., 2008)
Cohort	1 (8%)	(Grella & Rodriguez, 2011)
Retrospective	1 (8%)	(Farrell-Macdonald et al., 2014)
Study population		
Female only	10 (83%)	(Chan et al., 2005; Covington et al., 2008; Farrell-Macdonald et al., 2014; Grella & Rodriguez, 2011; Guydish et al., 2011; Messina et al., 2006; Miller et al., 2016; Nyamathi et al., 2018; Schram & Morash, 2002; Scott et al., 2017)
Mixed-gendered	2 (17%)	(J. E. Johnson et al., 2011; Needels et al., 2005)
Participants		
Total participants	4865	
Total women	3799	
Intervention participants (women)	2174	
Control participants (women)	1580	
Participants age		
Mean range	30.1–39.1	(Chan et al., 2005; Covington et al., 2008; Grella & Rodriguez, 2011; Guydish et al., 2011; J. E. Johnson et al., 2011; Messina et al., 2006; Miller et al., 2016; Needels et al., 2005; Nyamathi et al., 2018; Schram & Morash, 2002; Scott et al., 2017)
Median	31–40	(Schram & Morash, 2002)
Identified substance use disorder		
100%	9 (75%)	(Covington et al., 2008; Farrell-Macdonald et al., 2014; Grella & Rodriguez, 2011; Guydish et al., 2011; J. E. Johnson et al., 2011; Messina et al., 2006; Miller et al., 2016; Nyamathi et al., 2018; Scott et al., 2017)
< 100% (range 74–96%)	3 (25%)	(Chan et al., 2005; Needels et al., 2005; Schram & Morash, 2002)
Incarceration history		
Have been in prison before this reception (% range)	55%–92%	(Chan et al., 2005; Covington et al., 2008; Farrell-Macdonald et al., 2014; Grella & Rodriguez, 2011; Guydish et al., 2011; Nyamathi et al., 2018; Schram & Morash, 2002; Scott et al., 2017)
Previous contact with the criminal justice system (mean range)	6.3–10.4	(J. E. Johnson et al., 2011; Messina et al., 2006; Miller et al., 2016; Needels et al., 2005)
Parenting characteristics		
Mother (% range)	63–82%	(Chan et al., 2005; Covington et al., 2008; Grella & Rodriguez, 2011; Guydish et al., 2011; J. E. Johnson et al., 2011; Schram & Morash, 2002; Scott et al., 2017)
Average number per women	1 (8%)	(Messina et al., 2006)
Median number per women	2 (17%)	(Chan et al., 2005)
Intervention delivery		
Post-release	6 (50%)	(Chan et al., 2005; Covington et al., 2008; Guydish et al., 2011; J. E. Johnson et al., 2011; Nyamathi et al., 2018; Scott et al., 2017)
Transitional	6 (50%)	(Farrell-Macdonald et al., 2014; Grella & Rodriguez, 2011; Messina et al., 2006; Miller et al., 2016; Needels et al., 2005; Schram & Morash, 2002)
Intervention		
Probation Case Management	2 (17%)	(Chan et al., 2005; Guydish et al., 2011)
Dialectical Behavioural Therapy–Corrections Modified	1 (8%)	(Nyamathi et al., 2018)
Recovery Management Check-ups	1 (8%)	(Scott et al., 2017)
Delaware County Transition	1 (8%)	(Miller et al., 2016)
Methadone maintenance treatment	1 (8%)	(Farrell-Macdonald et al., 2014)
Female Offender Treatment and Employment Program	1 (8%)	(Grella & Rodriguez, 2011)
Collaborative Behavioral Management	1 (8%)	(J. E. Johnson et al., 2011)
Women’s Integrated Treatment model	1 (8%)	(Covington et al., 2008)
Prison-based substance abuse program and community-based after-care	1 (8%)	(Messina et al., 2006)
HealthLink jail and community services	1 (8%)	(Needels et al., 2005)
Life Skills program	1 (8%)	(Schram & Morash, 2002)
Comparator		
Standard parole/probation	4 (33%)	(Chan et al., 2005; Guydish et al., 2011; J. E. Johnson et al., 2011; Scott et al., 2017)
Pre-release treatment group	3 (25%)	(Farrell-Macdonald et al., 2014; Messina et al., 2006; Needels et al., 2005)
No treatment control group	2 (17%)	(Farrell-Macdonald et al., 2014; Messina et al., 2006)
Control group not clear	2 (17%)	(Miller et al., 2016; Schram & Morash, 2002)
Health Promotion program	1 (8%)	(Nyamathi et al., 2018)
Program non-completers	1 (8%)	(Grella & Rodriguez, 2011)
Pre/post test scores	1 (8%)	(Covington et al., 2008)
Setting		
Pre-release	6 (50%)	(Farrell-Macdonald et al., 2014; Grella & Rodriguez, 2011; Messina et al., 2006; Miller et al., 2016; Needels et al., 2005; Schram & Morash, 2002)
Jail/prison	4 (33%)	(Farrell-Macdonald et al., 2014; Grella & Rodriguez, 2011; Miller et al., 2016; Needels et al., 2005)
Prison camp	1 (8%)	(Schram & Morash, 2002)
Therapeutic Community (in-prison but separate to general prison population)	1 (8%)	(Messina et al., 2006)
Post-release	12 (100%)	(Chan et al., 2005; Covington et al., 2008; Farrell-Macdonald et al., 2014; Grella & Rodriguez, 2011; Guydish et al., 2011; J. E. Johnson et al., 2011; Messina et al., 2006; Miller et al., 2016; Needels et al., 2005; Nyamathi et al., 2018; Schram & Morash, 2002; Scott et al., 2017)
Community based (outpatient)	10 (83%)	(Chan et al., 2005; Farrell-Macdonald et al., 2014; Grella & Rodriguez, 2011; Guydish et al., 2011; J. E. Johnson et al., 2011; Miller et al., 2016; Needels et al., 2005; Nyamathi et al., 2018; Schram & Morash, 2002; Scott et al., 2017)
Residential treatment facility (inpatient)	1 (8%)	(Covington et al., 2008)
Post-release setting not clear	1 (8%)	(Messina et al., 2006)
Intervention length		
Pre-release		
6–12 months	2 (17%)	(Needels et al., 2005; Schram & Morash, 2002)
13–24 months	1 (8%)	(Messina et al., 2006)
Pre-release length not reported	3 (25%)	(Farrell-Macdonald et al., 2014; Grella & Rodriguez, 2011; Miller et al., 2016)
Post-release		
< 3 months	1 (8%)	(Schram & Morash, 2002)
3–6 months	2 (17%)	(J. E. Johnson et al., 2011; Messina et al., 2006)
7–12 months	5 (42%)	(Chan et al., 2005; Covington et al., 2008; Guydish et al., 2011; Needels et al., 2005; Nyamathi et al., 2018)
13–24 months	1 (8%)	(Grella & Rodriguez, 2011)
> 24 months	1 (8%)	(Scott et al., 2017)
Pre-release length not reported	2 (17%)	(Farrell-Macdonald et al., 2014; Miller et al., 2016)
Intervention attributes		
Community case management	8 (67%)	(Chan et al., 2005; Grella & Rodriguez, 2011; Guydish et al., 2011; Miller et al., 2016; Needels et al., 2005; Nyamathi et al., 2018; Schram & Morash, 2002; Scott et al., 2017)
Gender-responsive	7 (58%)	(Chan et al., 2005; Covington et al., 2008; Grella & Rodriguez, 2011; Guydish et al., 2011; Nyamathi et al., 2018; Schram & Morash, 2002; Scott et al., 2017)
Referrals to services	7 (58%)	(Chan et al., 2005; Grella & Rodriguez, 2011; Guydish et al., 2011; Miller et al., 2016; Needels et al., 2005; Nyamathi et al., 2018; Scott et al., 2017)
Cognitive behavioural treatment	7 (58%)	(Covington et al., 2008; J. E. Johnson et al., 2011; Messina et al., 2006; Miller et al., 2016; Needels et al., 2005; Nyamathi et al., 2018; Schram & Morash, 2002)
Imbedded substance-use treatment	5 (42%)	(Covington et al., 2008; Grella & Rodriguez, 2011; J. E. Johnson et al., 2011; Messina et al., 2006; Nyamathi et al., 2018)
Imbedded MH and/or trauma services	3 (25%)	(Covington et al., 2008; Needels et al., 2005; Scott et al., 2017)
Vocational services	4 (33%)	(Grella & Rodriguez, 2011; Guydish et al., 2011; J. E. Johnson et al., 2011; Schram & Morash, 2002)
Drug substitution therapy	1 (8%)	(Farrell-Macdonald et al., 2014)
Housing support	1 (8%)	(Schram & Morash, 2002)
Recidivism term used		
Recidivism	8 (67%)	(Farrell-Macdonald et al., 2014; Grella & Rodriguez, 2011; Guydish et al., 2011; J. E. Johnson et al., 2011; Miller et al., 2016; Nyamathi et al., 2018; Schram & Morash, 2002; Scott et al., 2017)
Criminal activity	1 (8%)	(Covington et al., 2008)
Return to custody	1 (8%)	(Messina et al., 2006)
Incarcerated	1 (8%)	(Chan et al., 2005)
Criminal justice system involvement	1 (8%)	(Needels et al., 2005)
Recidivism measure		
Return to custody	8 (67%)	(Chan et al., 2005; Farrell-Macdonald et al., 2014; Grella & Rodriguez, 2011; J. E. Johnson et al., 2011; Messina et al., 2006; Nyamathi et al., 2018; Schram & Morash, 2002; Scott et al., 2017)
Re-arrest	4 (33%)	(Guydish et al., 2011; J. E. Johnson et al., 2011; Needels et al., 2005; Scott et al., 2017)
Conviction-free	1 (8%)	(Covington et al., 2008)
Reoffended	1 (8%)	(Miller et al., 2016)
Reoffending post-release as a result of:		
Probation/parole violation	3 (25%)	(Grella & Rodriguez, 2011; Miller et al., 2016; Needels et al., 2005)
Charge with a new crime	3 (25%)	(Grella & Rodriguez, 2011; Miller et al., 2016; Scott et al., 2017)
Type of crime (drug, property, violent crime, prostitution)	1 (8%)	(Scott et al., 2017)
Date of first arrest	1 (8%)	(Guydish et al., 2011)
Follow-up time point (post-treatment)		
0 months	5 (42%)	(Chan et al., 2005; Guydish et al., 2011; Messina et al., 2006; Schram & Morash, 2002; Scott et al., 2017)
3 months	1 (8%)	(Needels et al., 2005)
6 months	3 (25%)	(Covington et al., 2008; J. E. Johnson et al., 2011; Nyamathi et al., 2018)
12 months	1 (8%)	(Grella & Rodriguez, 2011)
Not reported	2 (17%)	(Farrell-Macdonald et al., 2014; Miller et al., 2016)
Outcomes		
Recidivism	12 (100%)	(Chan et al., 2005; Covington et al., 2008; Farrell-Macdonald et al., 2014; Grella & Rodriguez, 2011; Guydish et al., 2011; J. E. Johnson et al., 2011; Messina et al., 2006; Miller et al., 2016; Needels et al., 2005; Nyamathi et al., 2018; Schram & Morash, 2002; Scott et al., 2017)
Substance use	6 (50%)	(Chan et al., 2005; Covington et al., 2008; Guydish et al., 2011; J. E. Johnson et al., 2011; Needels et al., 2005; Scott et al., 2017)
Mental Health	4 (33%)	(Chan et al., 2005; Covington et al., 2008; Guydish et al., 2011; Nyamathi et al., 2018)
Treatment utilization	4 (33%)	(Chan et al., 2005; Guydish et al., 2011; Needels et al., 2005; Scott et al., 2017)
HIV risk behaviours	2 (17%)	(Needels et al., 2005; Nyamathi et al., 2018)
Social support	2 (17%)	(Chan et al., 2005; Guydish et al., 2011)
Trauma symptomology	1 (8%)	(Covington et al., 2008)
Willingness/plans to participate in aftercare	1 (8%)	(Grella & Rodriguez, 2011)
Treatment completion status	1 (8%)	(Grella & Rodriguez, 2011)
Child custody	1 (8%)	(Chan et al., 2005)
Coping behaviours	1 (8%)	(Nyamathi et al., 2018)
Client satisfaction	1 (8%)	(Covington et al., 2008)
Discriminatory beliefs	1 (8%)	(Nyamathi et al., 2018)
Desire for help	1 (8%)	(Nyamathi et al., 2018)
Survival time in the community	1 (8%)	(Farrell-Macdonald et al., 2014)
Treatment readiness	1 (8%)	(Nyamathi et al., 2018)
Time in treatment	1 (8%)	(Grella & Rodriguez, 2011)
Participation in pre-release treatment	1 (8%)	(Grella & Rodriguez, 2011)

The post-release setting of programs was predominantly community-based (outpatient care) (*n* = 10) (Chan et al., 2005; Farrell-Macdonald et al., 2014; Grella & Rodriguez, 2011; Guydish et al., 2011; J. E. Johnson et al., 2011; Miller et al., 2016; Needels et al., 2005; Nyamathi et al., 2018; Schram & Morash, 2002; Scott et al., 2017), with one study occurring in a residential treatment facility (inpatient) (Covington et al., 2008). Most studies included women only (*n* = 9) (Chan et al., 2005; Farrell-Macdonald et al., 2014; Grella & Rodriguez, 2011; Messina et al., 2006; Nyamathi et al., 2018; Schram & Morash, 2002), while one accepted women with their children (Covington et al., 2008) and two were mixed-gendered (J. E. Johnson et al., 2011; Needels et al., 2005). Seven studies reported parenting characteristics (Chan et al., 2005; Covington et al., 2008; Grella & Rodriguez, 2011; Guydish et al., 2011; J. E. Johnson et al., 2011; Schram & Morash, 2002; Scott et al., 2017), of which the proportion of mothers ranged from 63 to 82% (excluding two studies who reported the average (Messina et al., 2006) and the median (Chan et al., 2005) number of children in their population).

Recidivism was a primary outcome in half (50%) of the studies (Farrell-Macdonald et al., 2014; Messina et al., 2006; Miller et al., 2016; Needels et al., 2005; Nyamathi et al., 2018; Schram & Morash, 2002) and a secondary outcome in the remaining studies (50%) (Chan et al., 2005; Covington et al., 2008; Grella & Rodriguez, 2011; Guydish et al., 2011; J. E. Johnson et al., 2011; Scott et al., 2017). Other outcomes included: substance-use outcomes post-release (*n* = 6) (Chan et al., 2005; Covington et al., 2008; Guydish et al., 2011; J. E. Johnson et al., 2011; Needels et al., 2005; Scott et al., 2017); treatment utilization (*n* = 4) (Chan et al., 2005; Guydish et al., 2011; Needels et al., 2005; Scott et al., 2017) MH outcomes (*n* = 4) (Chan et al., 2005; Covington et al., 2008; Guydish et al., 2011; Nyamathi et al., 2018), trauma symptomology (*n* = 1) (Covington et al., 2008) and child custody (*n* = 1) (Chan et al., 2005). Follow-up of women post-treatment varied between studies. Five studies captured follow-up data between 3 and 12 months post-intervention (Covington et al., 2008; Grella & Rodriguez, 2011; J. E. Johnson et al., 2011; Needels et al., 2005; Nyamathi et al., 2018), whilst five studies had no further follow-up past completion of the intervention (Chan et al., 2005; Guydish et al., 2011; Messina et al., 2006; Schram & Morash, 2002; Scott et al., 2017) and two studies had unclear follow-up timeframes (Farrell-Macdonald et al., 2014; Miller et al., 2016).

### Program characteristics

The 12 included studies assessed 11 different programs, with two studies evaluating the same intervention (Chan et al., 2005; Guydish et al., 2011). All interventions were grouped as post-release (*n* = 6; 50%) (Chan et al., 2005; Covington et al., 2008; Guydish et al., 2011; J. E. Johnson et al., 2011; Nyamathi et al., 2018; Scott et al., 2017) or transitional (*n* = 6; 50%) (Farrell-Macdonald et al., 2014; Grella & Rodriguez, 2011; Messina et al., 2006; Miller et al., 2016; Needels et al., 2005; Schram & Morash, 2002) programs (Table 1, and Table A1). One study observed the effects of methadone maintenance treatment (MMT) on opioid addicted participants (Farrell-Macdonald et al., 2014), the rest of the programs were non-pharmacological (*n* = 11) (Chan et al., 2005; Covington et al., 2008; Grella & Rodriguez, 2011; Guydish et al., 2011; J. E. Johnson et al., 2011; Messina et al., 2006; Miller et al., 2016; Needels et al., 2005; Nyamathi et al., 2018; Schram & Morash, 2002; Scott et al., 2017). The most common intervention attributes were community case management (*n* = 8) (Chan et al., 2005; Grella & Rodriguez, 2011; Guydish et al., 2011; Miller et al., 2016; Needels et al., 2005; Nyamathi et al., 2018; Schram & Morash, 2002; Scott et al., 2017), gender-responsive interventions (*n* = 7) (Chan et al., 2005; Covington et al., 2008; Grella & Rodriguez, 2011; Guydish et al., 2011; Nyamathi et al., 2018; Schram & Morash, 2002; Scott et al., 2017), and programs which used cognitive behavioural treatments (n = 7) (Covington et al., 2008; J. E. Johnson et al., 2011; Messina et al., 2006; Miller et al., 2016; Needels et al., 2005; Nyamathi et al., 2018; Schram & Morash, 2002). Seven studies (Chan et al., 2005; Grella & Rodriguez, 2011; Guydish et al., 2011; Miller et al., 2016; Needels et al., 2005; Nyamathi et al., 2018; Scott et al., 2017) referred women to services (SUD treatment, MH services, primary health care etc.) and seven had imbedded treatment services (SUD treatment (Covington et al., 2008; Grella & Rodriguez, 2011; J. E. Johnson et al., 2011; Messina et al., 2006; Nyamathi et al., 2018), MH/trauma services (Covington et al., 2008; Needels et al., 2005; Scott et al., 2017)). Other attributes included vocational services (*n* = 4) (Grella & Rodriguez, 2011; Guydish et al., 2011; J. E. Johnson et al., 2011; Schram & Morash, 2002) and one study provided housing support (Schram & Morash, 2002). The length of the post-release program varied across studies from 60 days (Schram & Morash, 2002) to three years (Scott et al., 2017) post-release, with the majority (42%) being between 7 and 12 months post-release (Chan et al., 2005; Covington et al., 2008; Guydish et al., 2011; Needels et al., 2005; Nyamathi et al., 2018). Two studies did not report intervention length (Farrell-Macdonald et al., 2014; Miller et al., 2016).

Comparison groups were diverse. Post-release programs (*n* = 6) were compared with usual care (standard probation/parole) in 67% of studies (Chan et al., 2005; Guydish et al., 2011; J. E. Johnson et al., 2011; Scott et al., 2017), with one of those studies (Scott et al., 2017) also conducting a within group review; one study (8.3%) compared to a another post-release program (Nyamathi et al., 2018) and one study (8.3%) compared participants on pre−/post-test scores (Covington et al., 2008). Transitional programs (n = 6) were compared to pre-release treatment groups in 50% of studies (Farrell-Macdonald et al., 2014; Messina et al., 2006; Needels et al., 2005) and two of those also compared to a no-treatment group (Farrell-Macdonald et al., 2014; Messina et al., 2006); two studies (33%) compared against a non-specific control group (Miller et al., 2016; Schram & Morash, 2002) and one study compared participant completers to non-completers (Grella & Rodriguez, 2011).

### Quality assessment

**Fig. 3:**
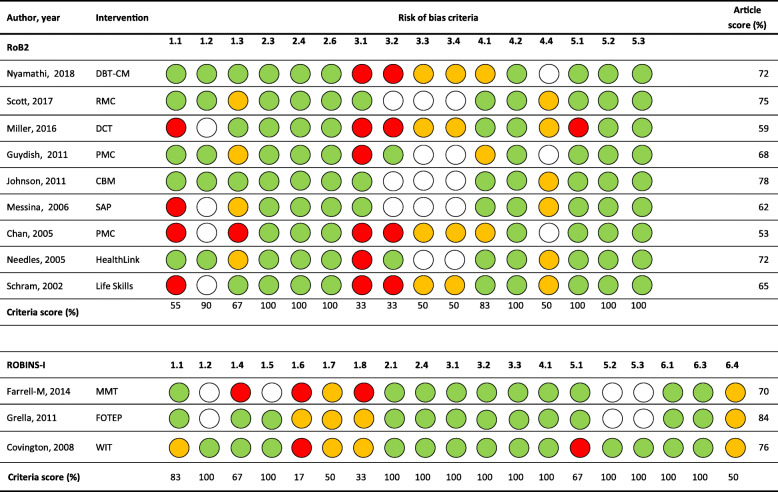
*Quality assessment heat map.* Note: CBM – Collaborative Behavioral Management; DBT-CM – Dialectical Behavioural Therapy–Corrections Modified; DCT – the Delaware County Transition; FOTEP – the Female Offender Treatment and Employment Program; MMT – methadone maintenance treatment; PCM – Probation Case Management; RMC – Recovery Management Check-ups; RoB2 – revised Cochrane Risk of Bias tool for randomized trials; ROBINS-I – Risk of Bias in Non-randomized Studies - of Interventions; SAP –prison-based substance abuse program and community-based after-care; WIT – the Women’s Integrated Treatment model

The overall quality of the included studies were of a fair quality, with an average score of 0.77 (range 0.53–0.84) (See Fig. 3). Individual criteria scores ranged from 17 to 100%. Missing or incomplete data was the lowest scoring item (RoB2 criteria 3.1 and 3.2, score 0.33). Many studies (58%) did not document reasons for participant drop-out (Chan et al., 2005; Covington et al., 2008; Guydish et al., 2011; Miller et al., 2016; Needels et al., 2005; Nyamathi et al., 2018; Schram & Morash, 2002), while a minority of control groups were not clearly described (17%) (Miller et al., 2016; Schram & Morash, 2002), intervention length and intensity not reported (25%) (Farrell-Macdonald et al., 2014; Grella & Rodriguez, 2011; Miller et al., 2016) and timeframes were unclear on when follow-up data was captured (17%) (Farrell-Macdonald et al., 2014; Miller et al., 2016). Allocation bias also scored low (RoB2 1.3, 0.67; ROBINS-1, 0.17), mainly due to major differences seen between groups at baseline (Chan et al., 2005; Covington et al., 2008; Guydish et al., 2011; Messina et al., 2006; Schram & Morash, 2002; Scott et al., 2017).

### Recidivism

The measure recidivism varied between studies and was used to quantify different crime-related events post-release (Table 2). A return-to-custody (RTC) was the most commonly used measure for recidivism (*n* = 8) (Chan et al., 2005; Farrell-Macdonald et al., 2014; Grella & Rodriguez, 2011; J. E. Johnson et al., 2011; Messina et al., 2006; Nyamathi et al., 2018; Schram & Morash, 2002; Scott et al., 2017). Recidivism was also a measure of re-arrest rates (*n* = 4) (Guydish et al., 2011; J. E. Johnson et al., 2011; Needels et al., 2005; Scott et al., 2017), the rate of reoffending (*n* = 1) (Miller et al., 2016) and being conviction-free at follow-up (n = 1) (Covington et al., 2008). Six studies used more than one measure for recidivism (Grella & Rodriguez, 2011; Guydish et al., 2011; J. E. Johnson et al., 2011; Miller et al., 2016; Needels et al., 2005; Scott et al., 2017), whereas six used a single measure (Chan et al., 2005; Covington et al., 2008; Farrell-Macdonald et al., 2014; Messina et al., 2006; Nyamathi et al., 2018; Schram & Morash, 2002). In total, five of six transitional studies (83%) reported significant reductions in reoffending compared to the control arm (Farrell-Macdonald et al., 2014; Grella & Rodriguez, 2011; Messina et al., 2006; Miller et al., 2016; Schram & Morash, 2002). Three post-release programs saw some effects: two had within group effects (Nyamathi et al., 2018; Scott et al., 2017); and one study reported reduced recidivism but lacked follow-up data to preclude significance (Covington et al., 2008).

**Table 2:** Study results

Author, year	Intervention vs comparator	Recidivism (term used)	Recidivism description	Follow-up time post-release (post-treatment)	Main source of outcomes data	Program attributes	Results	
							Recidivism	Health outcomes
*Post-release programs*								
Nyamathi, 2018	DBT-CM vs HP program	Recidivism	Recidivism was defined as responding “Yes” to the question “Have you been back to jail or prison within the past 6 months?”	9–15 months post-release (6 months)	Follow-up Interviews	1, 2, 3, 4, 5	*Recidivism*: Recidivism was reported among 15.5% of DBT-CM participants and 20.7% of HP participants (*p* = .0469). Among participants who recidivated, DBT-CM stayed in the community a mean ± SD days of 153 ± 80 compared to 86 ± 80 days for HP participants (*p* = 0.073) *Multivariable Analysis*: The reduction in recidivism in the DBT group was more pronounced in the model for participants age < 50 years (Model 2; *p* = 0.085) and the model for participants with Desire for Help score > 35 (Model 3; *p* = 0.050).	*Substance use*: The majority of the participants reported using drugs or alcohol during the 6 months prior to the interview (DBT-CM 69.2% and 67.7% HP)
Scott, 2017	RMC vs standard parole; and within treatment group: probation supervision vs. non-probation group	Recidivism	Recidivism was based on any subsequent arrest or incarcerations. The types of crimes included drug crime, property crime, prostitution, violent crime, and revocation of probation that resulted in a return to jail, arrest, or new charges.	Quarterly for 3-years post-release (0**)	Records data from Cook County Jail’s Incarceration Management and Cost recovery system and the State of Illinois’ Law Enforcement Agencies Data System, as well as self-reported data from the GAIN	1, 2, 3, 6	*Recidivism*: Total percentage of incarcerations from baseline to 36 months was 38% in the RMC group and 41% in the control group *Subject effects of probation supervision on recidivism*: Women in the probation group were more likely (i.e. worse) than those in the non-probation group on measures of new crimes (11% vs. 9%; *p* < 0.01); new arrests or incarcerations (25% vs. 12%; *p* < 0.01); and new crimes, arrests, or incarcerations (33% vs. 19%; *p* < 0.01). *Indirect effects of probation, self-help, and RMCs on recidivism*: Treatment in the previous quarter was positively related in the subsequent quarter to the likelihood of new crimes (OR = 1.76, *p* < 0 .01); new arrests or incarcerations (OR = 2.19, *p* < 0.01); and new crimes, arrests, or incarcerations (OR = 2.58, *p* < 0.01). Participation in intensive self-help activities in the previous quarter was also related to fewer new arrests and incarcerations (OR = 0.56, *p* < 0.05), crimes, arrests, or incarcerations (OR = 0.64, *p* < 0.05) in the next quarter. In addition, weekly alcohol and drug use was related to new crimes (OR = 2.54, *p* < 0.05); and new crimes, arrests, or incarcerations (OR = 1.28, *p* < 0.05). Finally, HIV risk behaviours were positively related to any new crimes (OR = 1.58, *p* < 0.05), but negatively related to new arrests or incarcerations (OR = 0.66, *p* < 0.05) and new crimes, arrests, or incarcerations (OR = 0.63, *p* < 0.05).	*Substance use*: NR *Experimental intervention effects of RMC (nested within probation status)*: RMCs had favourable effects on women in the community who were not on probation but no effect on those on probation – non-probation women (who were assigned to RMC) at the beginning of the quarter were more likely than the control group to engage in any days of substance-use treatment (8.9% vs. 4.5%, *p* < 0.01) and in more than 10 days of treatment (7.5% vs. 3.9%, *p* < 0.01). They were also less likely to engage in weekly alcohol and drug use (47% vs. 60%, *p* < 0.05), any unprotected sex (34% vs. 46%, *p* < 0.01), and any HIV risk behaviour (66% vs. 73%, *p* < 0.05). Among women on probation, none of these effects was present. *Indirect effects of probation, self-help, and RMCs*: treatment (in the previous quarter) was positively related in the subsequent quarter to weekly alcohol and drug use (*p* < 0.01). In contrast, 10 or more days of treatment (*p* < 0.05) and participation in self-help (*p* < 0.05) and intensive self-help activities (*p* < 0.05) predicted a lower likelihood of weekly alcohol and drug use.
Guydish, 2011	PCM vs standard probation	Recidivism	Number of arrests during the 12-month follow-up period and date of first arrest occurring in that period	6 and 12-months post-release (0)	San Francisco integrated court data management system	1, 2, 3, 7	*Recidivism*: The proportion arrested in the PCM group was 65.2% compared to 58.2% for standard probation (fisher’s exact = .364). *Number of total arrests*: Among those arrested at least once, the mean ± SD number of arrests was 3.45 ± 2.68 in the PCM condition and 3.26 ± 2.39 in standard probation (Mann–Whitney = 0.939). *Survival analysis*: mean time to first arrest was 7.26 ± 0.396 months (for PCM participants and 7.08 ± 0.369 months for those in standard probation.	*Substance use*: NR *Risk of substance-use*: PCM group has a 10% reduction in risk, relative to the standard probation, of being in the high alcohol severity category at 6 months (OR 0.90, *p* = 0.80), however there was a 41% increase in risk at 12 months (OR 1.41, *p* = 0.40). Likewise, there was a 21% increased risk of being in the high severity drug severity group relative to standard probation (OR 1.21, *p* = 0.59) but at 12-months PCM has a 36% reduction (OR 0.64, *p* = 0.20) *Outcome analysis and change over time*: no group effects or group by time interactions were observed *Service Utilization*: There were no significant differences between groups, at either time point or for any service measured *Delivery of the PCM Intervention and Exposure Analysis*: At 6 months, 53.6% of PCM and 11.6% of standard probation participants reported having seen their PO (face-to-face meeting, one or more times) (Fisher’s exact, *p* < 0.0001). At 12 months, the proportions were 43.4% and 8.5% (Fisher’s exact, *p* < 0.0001). In the exposure analysis, participants who reported seeing a PO two or more times during 6–12 months were more likely to be in the lower drug severity category both at 6 and 12 months (*p* = 0.0015). The time by case management interaction (*p* = 0.74) shows that this effect did not vary by time. Participants who reported seeing a PO two or more times during the period from 6 to 12 months were more likely to be in the lower social severity category at both 6 and 12 months (*p* = 0.0366). The time by case management interaction (*p* = 0.63) shows that this effect also does not vary by time. *Children (n = 100)*: 14.6% and 15.4% of mothers in PCM and standard probation reported living with their children in the past 30 days at the 6 month follow-up. At the 12 month follow-up 16.7% and 7.5%, respectively, reporting living with their children in the past 30 days. At 6-months, 23.5% and 20.8% participated in parenting classes in the past 6 months. At 12 months 20.8% and 17% participated in parenting classes between 6 and 12 months post-release. Even less received counselling about reunification, with 10.4% and 7.8% during the first 6 months post-release and 15.1% and 13.2% between 6 and 12 months post-release.
Johnson, 2011	CBM vs standard parole	Recidivism	Arrests, and reincarceration on a daily basis during the follow-up period	3 and 9-months post-release (6 months)	Timeline Follow-back calendar interview	4, 5, 7	*Recidivism*: CBM did not significantly reduce re-incarceration risk – 29% of the control participants and 21% of the CBM participants were re-incarcerated during the 9-month follow-up.	*Substance use*: 17% of control participants and 11% of CBM participants used their primary drug at any time during the 9 months post-release. When asked about alcohol only, 29% of the control participants and only 5% of the CBM participants used alcohol during this time.
Covington, 2008	WIT model pre/post test	Criminal activity	Percentage of clients who successfully completed the program who reported remaining conviction-free at follow up	Intake, 45-days, completion of HWR and BT, and exit (6 months)	Standardized assessment and program intake form responses	2, 4, 5, 6	*Recidivism*: 99% of the participants who successfully completed the program (*n* = 40–44) reported remaining conviction-free during the program. Of those who completed the six-month follow-up (*n* = 29), 97% reported not having a new conviction.	*Substance use*: 99% of the participants who successfully completed the program (n = 40–44) reported remaining drug and alcohol free during the program. Of those who completed the 6-month follow up (n = 29), 72% reported not using any alcohol or other drugs since exiting the program. *Children*: 79% had dependent children (age < 18). Of the 157 women, 52% planned to bring their children with them to the program and 22% reported being pregnant at the time of intake. *Health outcomes*: Of the 41 women who completed all three assessments, the average TSC-40 score at 45 days was 26.3 ± 20.4 and decreased to a mean score of 19.3 ± 19.2 after completion of HWR (*p* < 0.01). The scores continued to decrease to a mean of 17.5 ± 21.0 after completion of BT. Two subscale scores showed significant improvement between the 45-day time point and the completion of HWR: the mean subscale score of depression was 6.1 ± 4.6 at the 45-day point and 4.3 ± 4.7 after completion of HWR (*p* < 0.01) and the mean subscale score of sleep disturbances was 6.3 ± 5.4 and 4.3 ± 4.7 (*p* < 0.01). Anxiety and dissociation significantly lowered between 45 days and completion of BT (*p* < 0.05). While depression and sleep disturbances continued to improve with the completion of BT (*p <* 0.05). Mean BDI scores significantly decreased for the 186 study clients (program intake) 13.8 ± 9.3 to 45 days 10.4 ± 8.7 (*p* < 0.05). In addition, scores for those clients who completed an assessment at 45 days (10.2 ± 9.4), at completion of HWR (7.4 ± 8.2), and at the end of BT (4.5 ± 6.4) showed significant decreases at completion of each treatment component (*p* < 0.05)
Chan, 2005	PCM vs standard parole	Incarcerated	Incarcerated in the 30 days preceding interview at baseline, 6 months and 12-months.	6 and 12-months post-release (0)	Follow-up Interviews	1, 2, 3	*Recidivism*: Incarceration at 6 months was 46% for PCM and 55.9% for standard probation and was 49.1% and 50% at 12 months, respectively.	*Substance use*: NR *Risk of substance-use*: PCM group has a 7% increase in risk, relative to the standard probation, of being in the high alcohol severity category at 6 months (OR 1.07. *p* = 0.90), however there was an 8% decrease in risk at 12 months (OR 0.92, *p* = 0.88). PCM group has a 84% increased risk of being in the high severity drug severity group relative to standard probation (OR 1.84, *p* = 0.31) but at 12-months again a 8% reduction (OR 0.92, *p* = 0.88) *Parenting classes*: 32.6% and 19.4% of PCM and standard probation groups enrolled in parenting classes in the past 6 months. Between 6 and 12 months 8.2% PCM and 23.3% standard probation enrolled in parenting classes, neither time-point reached significance
*Transitional programs*								
Miller, 2016	DCT vs control group	Recidivism	Re-offending after being released from incarceration. Three recidivism variables were collected: 1. probation violation, 2.charged with a new crime, or 3. whether the participant was found to have recidivated with either a probation violation or a new crime	NR (NR)	Survey responses	1, 3, 4	*Recidivism*: New charge recidivism was reported among 15.6% DCT and 16.7% control group; Probation revocation recidivism among 28% and 57% (*p* < 0.05), respectively; and any recidivism among 31% vs. 70%; (*p* < 0.01) *Multivariate logistic regression models predicting the odds of new charge recidivism, probation revocation, and any recidivism*: women in the treatment group were significantly less likely to experience any recidivism relative to control group (*p* = 0.01). Being married were also marginally less likely to experience any recidivism (*p* = 0.05).	*Substance use*: NR
Farrell-MacDonald, 2014	MMT-continuing vs 1.terminated treatment and 2.no treatment group	Recidivism	RTC following release from prison, while under community supervision	27 months (NR)	CSC’s Offender Management System	8	*Recidivism*: 20% of the MMT-C, 52% of MMT-T, and 57% of the MMT-N group had a RTC. *Risk of an RCT*: indicates that the MMT-C group had a 65% lower risk of RTC than the MMT-N group (HR 0.35, CI 0.13–0.90). The risk of RTC for the MMT-T and MMT-N groups was not significantly different *Type of recidivism*: The majority of RTCs in each group (60% in MMT-C, 80% in MMT-T, 72% in MMT-N) was related to technical revocation.	*Substance use*: NR
Grella, 2011	FOTEP completers vs non-completers	Recidivism	Any RTP (for parole violation or a new charge) in California over 12 months.	18–27 months post-release (12 months)	CDCR’s OBIS	1, 2, 3, 5, 7	*Recidivism*: 36.8% of FOTEP participants RTP within 12 months of FOTEP discharge. Of those, FOTEP completers were less likely to RTC compared to non-completers at 12-months post-release follow-up (10.6% compared to 89.4%; *p* = 0.0001). *Type of recidivism*: A majority of all cases were RTC for a parole violation (64%), 22% were returned with a new term, and the remainder (14%) returned pending parole revocation. *Recidivism characteristics*: Individuals in the younger age groups (compared to older) had proportionately higher rates of RTP. A larger proportion of individuals whose primary commitment offense was property-related crime RTP, whereas a smaller proportion of those with drug-related offenses RTP, as compared with individuals with violent or other types of offenses. *Survival analysis on RTP at 12 months following discharge from FOTEP*: There is a direct linear relationship between time in treatment and risk of RTP, with increasing amounts of time in treatment associated with decreasing risk of RTP (*p* < 0.001). Other variables that were associated with RTP were region of parole, with participants in Region III about 25% less likely to RTP than those in Region I (*p* < 0.05). Individuals who participated in an in-custody treatment program prior to their admission to FOTEP were about 25% less likely to RTP compared with those who had not (*p* < 0.01). Individuals who completed FOTEP treatment were about 80% less likely to RTP within 12 months as compared with non-completers (*p* < 0.0001).	*Substance use*: NR *Motivation for treatment*: Higher motivation for treatment was associated with having a child in the welfare system, having been in prior drug treatment, and using “harder” drugs (i.e., cocaine, meth, opiates) rather than marijuana or alcohol, as one’s primary substance. Individuals who had been incarcerated more than once were marginally more likely to have higher motivation for treatment. Lower motivation for treatment was associated with being African American, Hispanic, or of “other” race/ethnicity, as compared with being White; and with parole region.
Messina, 2006	SAP + community after-care vs 1. SAP only and 2. no treatment	RTC	Percentage participants who RTC within six months following release to parole.	6 and 12-months post-release (0)	CDCR’s OBIS	4, 5	*Recidivism*: six month RCT rates for SAP participants were 21%, SAP and aftercare 6% and no treatment 16% (*p* < 0.05) *Multivariate findings*: A RTC within 6-months of parole was significantly associated with age and number of prior incarcerations. For each additional year in age, the odds of a 6-month RTC were decreased by 6.7% (*p* < 0.01). In contrast, for each additional incarceration, the odds of a six-month RTC were increased by 21.2% (*p* < 0.01). Total number of months in aftercare treatment approached significance (*p* < 0.06). A RTC within 12 months of parole was significantly associated with total number of prior incarcerations and total number of months in community-based aftercare. For each additional incarceration, the odds of a 12-month RTC were increased by 29.8% (*p* < 0.01). For each additional month in aftercare treatment, the odds of a 12-month RTC were reduced by 1.5% (*p* < 0.06). Prison-based treatment/no treatment status approached significance (*p* < 0.08).	*Substance use*: NR
Needels, 2005	HealthLink JC vs J only	Criminal Justice System involvement	Rearrests or parole violations	15-months post-release (3 months)	Follow-up Interviews	1, 3, 4, 6	*Recidivism*: Events resulting from activity after release from jail, Arrested – 39% JC group and 35.3% J-only; Had serious arrest charge – 1% and 4.1% (*p* < 0.05), respectively; Had drug charge – 19.5% and 18.4%; Convicted on at least one charge – 27.1% and 20.3%; Sentenced to incarceration – 21.4% and 15.5%; Served incarcerated time – 35.5% and 32.6%	*Substance use*: 40.4% of JC group self-reported drug-use (any) post-release, with 35.5% reporting hard drugs and 14.5% reporting marijuana. Similarly, 37.8% of J-only group self-reported any drug, 31.6% hard drugs and 18.6% marijuana. Crack/cocaine hair test results show that 26.4% of JC group and 29.1% J-only group had a negative test; 39.2% and 37.6% positive test, respectively; 1.1% and 0.8% unable to test; finally 33.3% and 32.5% unable to obtain hair sample. *Post-release treatment utilization*: 60% of JC participants met with their caseworkers after release; 51% of JC participants had contact with their caseworkers at least 6 months post-release; and 36% maintained contact for most or all of the 12-month eligibility period. In contrast, J-only participants were not eligible for post-release services. Caseworkers recorded an average of 6.5 h of contact during the 12-month period immediately after release, either directly with each female client or with a friend, family member, or service provider on behalf of the client. *Drug treatment:* JC participants (66%) were more likely than J-only participants (56.6%) to participate in drug treatment programs (*p* < 0.05), including the ones that provided services other than detoxification (64.4% and 53.3%, respectively) (*p* < 0.05). *HIV Risk*: There were no reductions in clients’ self-reported behaviours associated with risk of HIV infection
Schram, 2002	Life Skills program vs comparison group	Recidivism	Woman, who had been released for 60 days, could be designated into one of four statuses: 1. Not returned to a correctional facility; 2. returned to a correctional facility; 3. Still in a release center or on electronic monitoring or; 4.terminated*.	Baseline and 60-days post-release (0)	Survey responses and the Department of Corrections	1, 2, 4, 7, 9	*Recidivism*: 10% of Life Skills participants returned to the correctional system for violation of parole or new offences in the sixty day period after release compared to 25% for comparison group (*p* = 0.005).	*Substance use*: NR *Group Differences in Life Skills*: the only significant differences between groups on post-test scores were Powerful Others (*p* = 0.058); Cognitive dimension of the Coping (*p* = 0.03). *Differences in post-tests scores within treatment group*: Treatment group participants were more likely to use cognitive (*p* < 0.001), social (*p* < 0.001), spiritual (*p* = 0.024) and overall coping resources (*p* = 0.047) to handle stress than they had prior to program participation. Family health and nutrition: the analyses comparing the treatment group’s pre/post-test scores resulted the participants being significantly more confident on the post-test score that they could provide nutritious meals (*p* = 0.029).

Note: BDI – the Beck Depression Inventory; BT – Beyond Trauma; CBM – Collaborative Behavioral Management; CDRC – California Department of Corrections and Rehabilitation; CSC – The Correctional Service of Canada; DBT-CM – Dialectical Behavioural Therapy–Corrections Modified; DCT – the Delaware County Transition; FOTEP – the Female Offender Treatment and Employment Program; GAIN – Interviews responses from the modified version of the Global Appraisal of Individual Needs; HealthLink JC –Jail and community services; HealthLink J-only – jail services only; HP – Health Promotion program; HWR – Helping Women Recover; MH – mental health; MMT-C/T/N – methadone maintenance treatment-continued/terminated/no treatment; NR – not reported; OBIS – Offender Based Information System; OR – odds ratio; PCM – Probation Case Management; PO – probation/parole officer; RMC – Recovery Management Check-ups; RTC/P – return to custody/prison; SAP + aftercare –prison-based substance abuse program and community-based after-care; SAP only - prison-based substance abuse program only (pre-release); TSC-40 – the Trauma Symptom Checklist; WIT – the Women’s Integrated Treatment model; 1 - Community case management; 2 –Gender responsive; 3 –Referrals to services; 4 – Cognitive behavioural treatment; 5 – Imbedded substance-use treatment; 6 – Imbedded mental health and/or trauma services; 7 – Health promotion initiatives; 8 –Drug substitution therapy; 9 - Housing support

Table 3 visually breaks down study characteristics and the correlation between recidivism outcomes. Of which five/eight (62.5%) incorporated community case management (Grella & Rodriguez, 2011; Miller et al., 2016; Nyamathi et al., 2018; Schram & Morash, 2002; Scott et al., 2017); five/seven (71.4%) reported being gender-responsive (Covington et al., 2008; Grella & Rodriguez, 2011; Nyamathi et al., 2018; Schram & Morash, 2002; Scott et al., 2017); six/seven programs (85.7%) either included or referred participants to treatment services that targeted SUDs, MH and trauma (Covington et al., 2008; Grella & Rodriguez, 2011; Messina et al., 2006; Miller et al., 2016; Nyamathi et al., 2018; Scott et al., 2017) and five/seven (71%) used cognitive behavioural therapies (Covington et al., 2008; Messina et al., 2006; Miller et al., 2016; Nyamathi et al., 2018; Schram & Morash, 2002) (Tables 2 and 3). The length of the post-release component of the program (treatment in the community) varied from 60 days (Schram & Morash, 2002) to three years (Scott et al., 2017). Two studies did not report the post-release treatment length (Farrell-Macdonald et al., 2014; Miller et al., 2016).

**Table 3:** Program matrix to visually depict attributes correlating to outcome change

		Program attributes											Outcomes (S = significant and P = promising findings)	
Author, year	Intervention vs comparator	Post-release Intervention length	Follow-up post-treatment	1	2	3	4	5	6	7	8	9	Recidivism	Substance-use
*Post-release programs*														
Nyamathi, 2018	DBT-CM vs HP	3-9 m	6 m	X	X	X	X	X					P	
Scott, 2017	RMC vs Standard parole	3y	nil	X	X	X			X				P	P
Guydish, 2011	PMC vs standard probation; and RMC on supervision and not on supervision	12 m	nil	X	X	X				X				
Johnson, 2011	CBM vs standard parole	12 weeks	6 m				X	X		X				S
Covington, 2008	WIT pre/post test	12 m	6 m		X		X	X	X				P	P
Chan, 2005	PMC vs standard parole	12 m	nil	X	X	X								
*Transitional programs*														
Miller, 2016	DCT vs control group	NR	NR	X		X	X						S	
Farrell-MacDonald, 2014	MMT-C vs MMC-T and MMC-N	NR	27m*								X		S	
Grella, 2011	FOTEP completers vs FOTEP non-completers	6-15 m	12 m	X	X	X		X		X			S	
Messina, 2006	SAP + aftercare vs 1. SAP only and 2. no treatment	6 m	6 m				X	X					S	
Needels, 2005	HealthLink JC vs J-only	12 m	3 m	X		X	X		X					
Schram, 2002	Life Skills program vs comparison group	60-days	0	X	X		X			X		X	S	

Note: CBM – Collaborative Behavioral Management; DBT-CM – Dialectical Behavioural Therapy–Corrections Modified; DCT – the Delaware County Transition; FOTEP – the Female Offender Treatment and Employment Program;; HealthLink JC –Jail and community; HealthLink J-only – jail services only; HP – Health Promotion program; m – months; MMT-C/T/N – methadone maintenance treatment-continuing/terminated/no treatment; nil – no follow-up past completion of the post-release program; NR – not reported; P – authors concluded promising findings but results were not statistically significant; PMC – Probation Case Management; RMC – Recovery Management Check-ups; S – results were statistically significant; SAP + aftercare –prison-based substance abuse program and community-based after-care; SAP only - prison-based substance abuse program only (pre-release); WIT – the Women’s Integrated Treatment model; y – years; 1 – Community case management; 2 – Gender responsive; 3 – Referrals to services; 4 – Cognitive behavioural treatment; 5 – Imbedded substance abuse treatment; 6 – Imbedded MH and/or trauma services; 7 – Health promotion initiatives; 8 – Drug substitution therapy; 9 – Housing support

*Farrell-MacDonald reported that follow-up data was collect 27 months post-release but as they were unclear on intervention length we do not know how long post-treatment the follow-up data was collected. For this we reported 27 months for post-treatment

### Substance use

Six studies (50%) (Chan et al., 2005; Covington et al., 2008; Guydish et al., 2011; J. E. Johnson et al., 2011; Needels et al., 2005; Scott et al., 2017) examined the effect of the program on substance-use post-release, of which five (83%) were post-release programs (Chan et al., 2005; Covington et al., 2008; Guydish et al., 2011; J. E. Johnson et al., 2011; Scott et al., 2017) and one (17%) was transitional (Needels et al., 2005) (Tables 2 and 3). Three post-release programs (50%) reported reduced substance-use at follow-up (Covington et al., 2008; J. E. Johnson et al., 2011; Scott et al., 2017). One program (J. E. Johnson et al., 2011) reported that participants in the intervention group significantly reduced substance-use post-release; one study had within group effects (Scott et al., 2017) and another study (Covington et al., 2008) saw reductions but lacked follow-up data to preclude significance. The attributes that supported these programs included SUD, MH and trauma treatment services (100%) (Covington et al., 2008; J. E. Johnson et al., 2011; Scott et al., 2017); two programs (66.7%) were gender-responsive (Covington et al., 2008; Scott et al., 2017), two (66.7%) had community case management (Covington et al., 2008; Scott et al., 2017) and two (66.7%) used cognitive behavioural therapies (Covington et al., 2008; J. E. Johnson et al., 2011). No correlation between reduced substance-use and recidivism post-release was seen.

## Discussion

This is the first systematic review to examine post-release and transitional programs offered to women with SUDs exiting prison to the community. In total we found 12 articles, which examined 11 programs, dating back to 2002, all conducted in North America. The objective of this review was to highlight the evidence about the effectiveness of post-release and transitional programs offered to women with SUDs and reveal what program attributes were common among successful programs.

The preliminary findings suggest that transitional programs had greater effects at reducing recidivism compared to post-release alone (83% compared to 50%). A major benefit for transitional programs is the continuity of care from prison to the community. Transitional support has been previously shown to assist participants in retaining rehabilitative health gains and reducing the risk of injury and death which is high for women with SUDs post-release (Abbot, Magin, Lujic, & Hu, 2017; Feild, 1998; MacDonald, Williams, & Kane, n.d.; Sullivan et al., 2019). Furthermore, as previously discussed, qualitative data also supports the use of transitional programs, as they facilitate pre-release linkage to health and social services in the community.

We were unable to make any correlations between substance-use and recidivism due to a limited pool of studies that reported substance-use as an outcome (RQ2). This is problematic considering all studies included women with SUDs and the direct correlation between substance-use and criminal offending for women is well understood (Fearn et al., 2016; H. Johnson, 2006). A major strength of this study is that it was the first to review and explore a variety of post-release and transitional programs for women with SUDs. As a result, we were able to critically examine the specific attributes of each program and make correlations between those attributes and improved post-release outcomes. Future research in this area can design or incorporate our findings into their interventions to further improve post-release outcomes for women exiting prison.

Five programs reported that allocation to the intervention group significantly reduced recidivism compared to the control group and another three concluded promising effects (RQ1). The attributes that contributed to the success of these programs were transitional, gender-responsive interventions which provided individualised support through community case management, with the use of cognitive behavioural therapies, as well as having substance-use, MH and trauma services available (whether it was imbedded, or women were referred to external services) (RQ3). Six studies reviewed substance-use post-release, and of those, three reported reduced substance use among program participants (RQ1). Reductions in substance-use was associated with programs that offered gender-responsive support, used cognitive behavioural therapies, and provided substance-use treatment, MH and trauma services (imbedded or referred) (RQ3).

These findings reinforce the existing evidence that the design of transitional programs need to address criminogenic risk factors of women in prison (Borzycki, 2005; Borzycki & Baldry, 2003; Carlton & Segrave, 2016) and indicates the benefit of programs tailored to these characteristics and needs. However, we cannot determine from these studies the specifics of what was delivered to women under the banner of ‘individualised support through community case management’ or ‘gender-responsive’ interventions. In this review all studies that incorporated community case management included the role of a case manager who provided individualised links between women and external community-based services. There was no clear identification of what services women prioritised, were referred to, or managed to attend, nor the duration of attendance. Case management is the coordination of health and social services for a particular person. When employed effectively, it can bridge the services received inside prison and connect clients to appropriate community services, improving interagency information-sharing and continuity of care for individual clients (Corrective Services NSW, 2017; Feild, 1998; Warwick, Dodd, & Neusteter, 2012). The flow on effects of improved wellbeing and rehabilitation results in increased survival-time in the community, improved health outcomes including substance-use, which ultimately improves recidivism rates for participants. These preliminary results support the use of community case management. However, further high evidence trials that clearly describe and measure the services women are referred to are needed to continue to build on the evidence pool for women exiting prison with an SUD.

Similarly, many programs described their intervention as gender-responsive without any further description of what that involved. It should be noted that gender-responsive programming must include creating an environment through site and staff selection, and program development, content and material that reflects an understanding of the realities of the lives of women in criminal justice settings and addresses their specific challenges and strengths (Covington & Bloom, 2006). In this review, five out of seven gender-responsive studies had an impact on recidivism. In addition, one study (Miller et al., 2016) provided community case management but did not state whether it was gender-responsive or not. It could be argued that case management is gender-responsive as it provides individualised support by linking to services based on an individual needs assessment which would target criminogenic needs, which should therefore be based on gender. This highlights two main points: 1) clearer reporting is required on what is provided when an intervention is described as gender-responsive or including case-management; and 2) Gender-responsive approaches are important and we need further research to extrapolate the aspects of gender-responsive programs that are helpful to women.

Findings from quantitative studies have shown specific attributes associated with post-release success, qualitative literature suggests there are other essential program characteristics not discussed in this review. Incarcerated women and service providers who work directly with women exiting prison have reported that stable housing, employment and family-related needs are the most critical attributes to post-release success for women (Kendall, Redshaw, Ward, Wayland, & Sullivan, 2018; O’Brien P. & Leem N., 2007; B. E. Salem et al., 2013). In our systematic review, no studies measured employment and housing status, or child custody in the follow-up periods. Furthermore, qualitative studies have identified the importance of continuity of care, pre-release linkage and emphasised the importance of the relationship between service providers and women participants (J. E. Johnson et al., 2013; Kendall et al., 2018; O’Brien P. & Leem N., 2007; B. E. Salem et al., 2013). Whilst findings from our systematic review reinforce the evidence for transitional programs, they did not measure relational or acceptability aspects of program implementation. We suggest that future interventions involve key stakeholders (e.g. women with SUDs and service providers) in the program design process to get a deeper understanding of what women not only need but what attributes they want to be included in a post-release program.

### Limitations

The current evidence suggests that women benefit from continuity of care from prison to the community, which incorporated gender-responsive programming and individualised case management. Generalisability is, however, limited by the fact that the majority of studies were conducted in the United States. It remains uncertain whether these programs will be effective with women in countries with a different social structure. Nevertheless, key program attributes are transferable and can inform program development.

The general scarcity of literature meant that we were unable to synthesise the true effectiveness of programs for women exiting prison with SUDs. A meta-analysis was not feasible due to the diverse range of included programs and methodological weaknesses including a lack of stringent study design and various chosen control groups, which in effect has impacted the ability to answer our research question with significance. Very few comparison groups were genuinely usual care or ‘no treatment’. In most cases, the control group was receiving another program, thereby making it impossible to isolate the impact of the program under investigation.

Further, understanding the long-term impact of programs is limited due to a lack of appropriate follow-up data. Five programs did not capture data past the completion of the program. Where changes were found, there are limits to how long these changes could be assumed to last due to a lack of proper long-term follow-up. More research is needed on the effectiveness of post-release programs for women. They need to be of rigorous study design, with appropriate control groups and follow-up to allow evaluation of program effectiveness.

Some studies failed to report program length, frequency of intervention, and follow-up time-points. It is important to clearly describe intervention modalities so that appropriate comparisons can be made. Unexplained lost to follow-up was common among studies, with no detail on important outcomes such as program dropout, accommodation change, homelessness, rearrested/reincarceration, hospitalisation, or death. All critical to understanding the effectiveness of an intervention and recidivism. Follow-up timeframes are also an important indication of how well an intervention was able to influence participant actions post-release such as recidivism and substance-use. Many studies did not follow participants past the completion of the intervention not allowing measurement of long-term impact. A follow-up period of two years has been recommended by a number of researchers as being optimal to understand the long-term effects of a program on participants (Andersen & Skardhamar, 2015; Office of the Inspector of Custodial Services, 2014; Yukhnenko et al., 2019).

Recidivism is one of the most fundamental outcome measures used in criminal justice research (Duwe, 2017; King & Elderbroom, 2014; Leverentz, Chen, Christian, & Maruna, 2020; Urban Institute, n.d.). All studies in this review used recidivism to measure program success however, we found it was inconsistently measured and there was a lack of standardisation across studies. Another limitation relating to recidivism was that most studies used the term recidivism to express a single RTC event. This is a blunt measure, simplifying a complex series of events, failing to account for the legislative and policy context in which a RTC occurs. As a result, readers are given only a partial view of how the criminal justice system operates and the position of women within it. To make recidivism a more meaningful measure we must move beyond a single event that measures success/failure of a program. A series of events such as rearrest, reconviction and reincarceration post-release as well as desistence, time to arrest, offence type and severity (King & Elderbroom, 2014; Urban Institute, n.d.). This suite of measures provides a timeline of events to give readers and policy makers a clearer view of the post-release experience and challenges.

Accounting for the context of health and social disadvantage experienced by women in prison, utilising health and social measures is also required. Almost all women within each study reported having a SUD prior to incarceration, however only six studies reported substance-use post-release. Furthermore, MH, trauma, child custody, housing and employment outcomes were not analysed. This is concerning, considering the extensiveness of research illustrating these characteristics and their influence on health and recidivism post-release (Baldry, 2010; Carlton & Segrave, 2016; Langan & Pelissier, 2001; Sullivan et al., 2019). Future studies should include, or at least measure, these determinants in any future analysis to give a deeper understanding as to why a program was successful or not.

## Conclusion

There is a paucity of literature on the effectiveness of post-release programs for women exiting prison with a SUD and the studies available contain significant methodological and conceptual limitations. There is a breadth of research that outlines the differences of characteristics of men and women within the criminal justice system, however because women make up a small proportion of the total prison population, they have received limited research attention in comparison. Recidivism rates illustrate that remaining in the community after any period in prison is difficult for women with SUDs. The rising rates of women in prison is a serious health and social policy issue in the context of what is already known about the intersecting health and social inequality experienced by women in prison and the barriers to women accessing social determinants of health resulting from disempowerment within broader social structures. The results from this review indicate that transitional, gender-responsive programs that incorporate individualised community case management and target co-morbid MH and SUD can have a significant impact on post-release outcomes. Building upon these findings, development of programs for women transitioning back into the community should as a first step incorporate nuanced measures for recidivism and integrate the successful program attributes highlighted by this review.

## Supplementary Information

**Additional file 1.** Database search strategy.

**Additional file 2.** Included studies.

**Additional file 3.** Revised Cochrane risk-of-bias tool for randomized trials (RoB 2) TEMPLATE FOR COMPLETION.

**Additional file 4.** The Risk Of Bias In Non-randomized Studies – of Interventions (ROBINS-I) assessment tool.

## Data Availability

Not applicable.
