# Supplementary material for: A systematic review of post-release programs for women exiting prison with substance-use disorders: assessing current programs and weighing the evidence
Source: Health Justice. 2022 Jan 3;10:1. doi: 10.1186/s40352-021-00162-6 (PMC8725487; doi:10.1186/s40352-021-00162-6)
Supplement: Supplementary file 1 — Additional file 1. Database search strategy. [file 40352_2021_162_MOESM1_ESM.docx]

# Additional files

Additional file 1: Database search strategy

| Original search strategy: September 2019 | Updated search strategy: Feburary 2020 |
| --- | --- |
| (women OR woman OR female OR gender*) AND  (ex-convict* OR ex-offender* OR post-release OR postrelease OR parole* OR probation OR re-entry OR re-integration OR reintegration OR post-incarcer* OR diversion* OR throughcare) AND  (prison OR jail OR gaol OR custody OR ‘community corrections’ OR remand OR corrections OR incarcer* OR diversionary OR "drug court") AND  (intervention* OR program* OR treatment OR throughcare OR recovery OR services OR rehabilitat* OR “re-entry program*”) | (woman or women or female* or gender) AND  (ex-convict* or ex-offender* or post-release or postrelease or parole* or probation or re-entry or re-integration or reintegration or post-incarcer* or diversion* or throughcare) AND  (prison* or jail or gaol or custody or “community corrections” or remand or corrections or incarcer* or diversionary or "drug court") AND  (intervention* or program* or treatment or throughcare or recovery or services or rehabilitat* or “re-entry program*”) AND  (“substance use” or “substance abuse” or “drug* and alcohol*” or “drug use” or “drug abuse” or alcohol* or “drug dependen*” or addict*) AND  (recidivism or reoffend* or re-offend*) |
