# Supplementary material for: A systematic review of post-release programs for women exiting prison with substance-use disorders: assessing current programs and weighing the evidence
Source: Health Justice. 2022 Jan 3;10:1. doi: 10.1186/s40352-021-00162-6 (PMC8725487; doi:10.1186/s40352-021-00162-6)
Supplement: Supplementary file 2 — Additional file 2. Included studies. [file 40352_2021_162_MOESM2_ESM.docx]

Additional file 2: Included studies

| Author, year | Country, period | Study design | Setting | Target population (mean age ± SD or range) | Sample size  (%/n female) | Program | Length and intensity | Comparison group(s) | Primary and secondary outcomes | Quality assessment score |
| --- | --- | --- | --- | --- | --- | --- | --- | --- | --- | --- |
| *Post-release programs* | | | | | | | | | | |
| Nyamathi, 20181 | US, 2015-2016 | Pilot RCT | Community shelter-based drop-in sites and residential drug treatment programs | Aged 18 to 60 years; had used drugs prior to their latest incarceration; and were considered homeless prior to discharge from incarceration  (DBT-CM 39.1 ± 11.5; HP 38.6 ± 11.3) | N=130 (100%).  DBT-CM n=65; HP n= 65 | **Dialectical Behavioural Therapy–Corrections Modified (DBT-CM) program**: The DBT-CM sessions focused on reduction of drug and alcohol use and strategies to avoid or eliminate use, strategies for coping positively, and building a positive life despite the challenges the women experienced over a lifetime. The one-on-one sessions focused on discussing diary cards that the women wrote up weekly and discussion of challenges to meeting their targeted goals. Furthermore, as the DBT included a case management component, ongoing contact was encouraged on a weekly basis over the 6-month period where they helped with referrals (e.g., health care, housing, employment skills, etc.). | **Length**: 3-months minimum and up to nine months  **Intensity**: 6 group and 6 one-on-one sessions over a 3-month period. After this only DBT-CM participants had the option for weekly contact, over the next 6-month period. | **Health Promotion (HP) program**: six group and six one-on-one sessions over a 3-month period. The six HP group sessions, conducted over the 3-month period, were focused on chronic diseases: diabetes, heart disease, sexually transmitted infections, HIV/AIDS, parenting skills, and community and family reintegration. Participants had up to 12 weeks to complete the HP group sessions; however, after 12 weeks, there was no ongoing meeting of the participants in relation to referrals and ongoing support. | **Primary**: Recidivism  **Secondary**: Alcohol and drug use, coping behaviours, discriminatory beliefs, depressive symptomology, mental health index, posttraumatic stress disorder, anger and hostility, desire for help, treatment readiness | 0.57 |
| Scott, 2017 | US, 2008-2010 | RCT | Community | Aged 18 years and over; had an SUD; plans to live in Chicago within the next 12 months; fluent in English or Spanish; spent a minimum of >14 days incarcerated; and were being released to the community  (36.7 ± 10.4) | N=480 (100%).  RMCs n=238; Standard parole n=242 | **Recovery management check-ups (RMCs)***: RMC linkage managers connect clients to treatment and employ engagement and retention protocols to assist clients in obtaining long-term recovery care. The linkage managers used motivational interviewing techniques to address current substance use, HIV risk practices, or criminal activity; to discuss the barriers to desistance and strategies to refrain from those behaviours; and to assess participant’s motivation for change. During the first 90 days post-release, RMC group received a modified, gender-focused intervention to reduce their risk of HIV. The intervention consisted of an assessment of HIV risk behaviours, HIV knowledge, and condom self-efficacy; an introduction to HIV-related health conditions and health promotion strategies (assertive communication, self-empowerment, and avoidance of violent and other unsafe situations); referrals for substance abuse and HIV treatment; and the provision of male and female condoms. | **Length**: Three years  **Intensity**: Women met with linkage managers after completing each research interview at release: 30, 60, and 90 days post-release and quarterly thereafter for up to 36 months. | **Standard parole***: not clear; includes interviews monthly for 90 days and quarterly for 3 years post-release | **Primary**: treatment utilization (increasing the rate of treatment participation any and 10+ days)  **Secondary**: Self-help participation, HIV risk behaviours, criminal recidivism, and substance use problems and relapse. | 0.65 |
| Guydish, 2011 | US, 1998-1999 | RCT | Community  (Post-release) | Residents of the city and county of San Francisco; Aged 18 years or older, who had a SUD and were involved in the criminal justice system  (34.7 ± 9.2) | N=183 (100%)  PCM n=92; Standard probation n=91 | **Probation case management (PCM)**: PCM was differentiated from standard probation by (a) lower caseloads and increased contact with clients, (b) uniform client screening and assessment procedures, (c) therapeutic and advocacy orientation, and (d) referrals to health and human services. PCM officers had approximately 50 clients at any time. | **Length**: 12-months  **Intensity**: Minimum contact: twice per month for one year | **Standard probation**: officers typically supervised caseloads of 100 to 150 clients at any time. The general tasks of probation include the preparation of presentence investigations and reports for the courts, supervision of those on probation and enforcement of conditions of probation, and assisting offenders in finding needed services. | **Primary**: Addiction severity over time  **Secondary**: Psychiatric symptoms, social support, service utilization and arrest data | 0.61 |
| Johnson, 2011 | US, 2005-2008 | RCT | Community  (Post-release) | Aged 18 years or older; English speaking; probable SUD immediately prior to incarceration or mandated to drug treatment; substance use treatment as a mandated or recommended condition of parole; moderate to high risk of drug use relapse and/or recidivism (determined by a LCSF score of >7 or by a history of two or more prior episodes of drug abuse treatment or drug-related convictions  (35.6 ± 8.5) | N=431 (17.86%; n=77)  CBM n=39; Standard parole n=38 | **Collaborative Behavioral Management (CBM)**: In CBM, POs used positive tools to shape behaviour in a prosocial direction through the definition and reinforcement of incremental steps toward rehabilitation. CBM has four major elements: 1.It explicitly articulates the roles of both staff and offenders, their expectations of one another, and the consequences if offenders meet or fail to meet those expectations; 2. it negotiates a behavioural contract that specifies concrete target behaviours in which the offender is expected to engage on a weekly basis. These target behaviours include requirements of supervision and formal addiction treatment and involvement in behaviours that compete with drug use (e.g., getting a job, enhancing nondrug social network); 3. It regularly monitors adherence to the weekly behavioural contract and administers both reinforcers and sanctions to shape behaviour; 4. CBM establishes a systematic, standardised, and progressive approach to reinforcement and sanctioning to ensure consistency and fairness. | **Length**: 12 weeks  **Intensity**: Weekly sessions over 12 weeks | **Standard parole**: included, at minimum, face-to-face contacts and drug testing (random, observed). Typical parole supervision involves week to monthly in-person contacts between the offender and PO to improve compliance with conditions of release (e.g., treatment attendance and drug abstinence). In this study, average contacts between parolees and the PO ranged from 1 to 4 per month, as did frequency of required urine tests; all POs had an affiliation with an outpatient substance abuse treatment program, the type of treatment offered was cognitive–behavioural in four sites and limited to alcohol and drug education in two sites. | **Primary**: Yes/no use of primary drug and alcohol  **Secondary**: Prison recidivism | 0.68 |
| Covington, 2008 | US, 2004-2006 | One group pre-test/post-test | Residential treatment facility for women and women with children  (Post-release) | Women with SUDs were referred to the program from various programs (mandated or self-referrals) and those who completed each set of assessments and/or forms as they progressed through treatment  (30.1; range 18-54 years) | Completion of first 45 days N=202 (100%)***  Completion of baseline assessment n=188–192; Completion of HWR Forms n=82–89; Completion of BT Forms n=51–53; Completion of 45 days, HWR and BT Forms n=40–44; Completion of both Intake and Exit Forms n=79–84 | **The Women’s Integrated Treatment (WIT) model**: is a gender-responsive and trauma informed curricula that integrates trauma and substance abuse treatment. Participants completed Beyond Trauma (BT) after the successful completion of Helping Women Recover (HWR). HWR is an integrated program for treating addiction. It is a manualized curriculum for treating women with histories of addiction and trauma. The sessions are in four modules: Self, Relationships, Sexuality, and Spirituality. BT is also a trauma-specific, healing journey for women. The major emphasis is on coping skills, with specific exercises for developing emotional wellness. Three modules focused on violence, abuse and trauma, the impact of trauma, and healing from trauma | **Length**: 12-months  **Intensity**: Orientation is 45 day (pre-program), HWR included 17 sessions and BT included 33 sessions for each participant | NA – Women who successfully completed the programs were assessed both before and after completion of the programs | **Primary**: Trauma symptomology  **Secondary**: Current drug use and criminal activity, mental health, client satisfaction | 0.68 |
| Chan, 2005 | US, 1997-1998 | Quasi-experimental | Community  (Post-release) | Women were residents of the City and County of San Francisco, were 18 years or older, had a SUD, and were involved in the criminal justice system. In addition, women must have been willing to enter PCM if a slot was available and must have been willing to enter substance abuse treatment.  (PCM 31.8 ± 7.1; Standard probation 33.0 ± 7.6) | N=109 (100%)  PCM n=65; Standard probation n=44 | **Probation Case Management (PCM)**: Case managers were selected from among current POs and retained the role of PO as well as case manager. PCM was differentiated from standard probation by (a) lower caseloads and increased contact with clients, (b) uniform client screening and assessment procedures, (c) therapeutic and advocacy orientation, and (d) referrals to health and human services. PCM officers had approximately 50 clients at any time. Therapeutic and advocacy activities included gender-specific client education about addiction and counselling. Case management activities included attending treatment planning meetings, going to court or to the housing authority with the client, and making home visits/attending medical appointments. In addition to substance abuse treatment, referral resources available to case managers included health and mental health services, child care and child reunification services, educational and employment counselling, and assistance with housing needs. | **Length**: 12-months  **Intensity**: Minimum contact: twice per month for one year | **Standard probation**: In standard probation, officers typically supervised caseloads of 100 to 150 clients, although this could vary based on the specific program or type of caseload. The general tasks of probation include preparing presentence investigations and reports for the Courts, supervising offenders placed on probation, enforcing court-ordered conditions, and assisting offenders in finding treatment and other services | **Primary**: Substance use  **Secondary**: Psychiatric symptoms, social support and current incarceration, child custody and services received at each time point. | 0.41 |
| *Transitional programs* | | | | | | | | | | |
| Miller, 2016 | US, 2012-2014 | Quasi-experimental design | Jail and Community | Not clear  (30.7 ± 8.02; 18-63) | N=62 (100%).  DCT n=32; Control n=30 | **The Delaware County Transition (DCT)** **program**: an individualized treatment and case management intervention strategy. DCT program staff (one re-entry coordinator and one case manager) receive referrals to the program, meet with women individually and in groups, conduct assessments, assist women in developing an individualized Re-entry Accountability Plan, coordinate mental health, medical, and drug treatment, and link women to community resources pre-release. Recovery services, anger management, mental health, and other identified services are provided by partner agencies in the community. Re-entry case-management proactively engage women and coordinate stakeholders to effect therapeutic collaborations comprising a continuum of re-entry services toward the goals of enhancing the likelihood of offender success and safer communities following release. | **Length**: NR  **Intensity**: NR | **Control group**: not clear | **Primary**: Recidivism | 0.5 |
| Farrell-MacDonald, 2014 | Canada, 2003-2008 | Retrospective study | CSC** and Community | Women federal offenders who were initiated on MMT while incarcerated in a CSC facility and released into the community  (Age at admission – MMT-C 33.0 ± 7.2; MMT-T 34.5 ± 8.0; MMT-N 31.3 ± 7.4) | N=137 (100%).  MMT-C n=25; MMT-T n=67; MMT-N n=45 | **Methadone Maintenance Treatment-Continuing (MMT-C)**: Community-based urinalysis results for methadone were used – as the best available proxy indicator for post-release MMT involvement and status. Those with at least one methadone-positive test, for which legitimacy was confirmed (based on CSC’s classification of the positive urinalysis test being due to a prescribed medication), were classified as offenders who were continuing MMT post-release. | **Length**: Pre-release: NR; Post-release: NR  **Intensity**: Pre-release: NR; Post-release: NR | **Methadone Maintenance Treatment=Terminated (MMT-T)**: Those with methadone-negative urine tests, or illegitimate methadone-positive results, post-release  **Methadone Maintenance Treatment=no treatment (MMT-N)**: offenders with a moderate to severe opioid substance problem but did not participate in MMT while incarcerated | **Primary**: Return to custody  **Secondary**: Number of days in community supervision | 0.61 |
| Grella, 2011 | US, 2005-2007 | Cohort study | Prison and community-based aftercare | Women parolees with a history of substance-use problems  (36.6 ± 8.7) | N=1182 (100%)  RTP-no n=747; RTP-yes n=435 | **The Female Offender Treatment and Employment Program (FOTEP)**: The core components consist of residential drug abuse treatment, comprehensive case management, vocational services, and parenting-related services. | **Length**: Pre-release: NR; Post-release: 6-15 months  **Intensity**: Pre-release: NR; Post-release: NR | **FOTEP**: Participants who did not complete treatment | **Primary**: Willingness and plans to participate in aftercare  **Secondary**: Recidivism, completion status, time in treatment (in days), and participation in an in-custody treatment program (prior to parole) | 0.74 |
| Messina, 2006 | US, 2004 | Quasi-experimental non-randomised control trial | In-prison TC; post-release setting not clear | Inmates who have a documented history of a SUD, who have between 6-24 months left to serve on their current sentence  (35.8) | N=316 (100%)  SAP n=171; No treatment n=145 | **Prison-based substance abuse program and community-based after-care (SAP + aftercare)**: SAP was available at the end of inmates' prison sentence. Characteristics of the prison based SAP include: (1) activities that embody positive values that start a process of socialization; (2) treatment staff who provide positive role models (many are recovering addicts themselves); (3) an alternative concept of inmates that is usually much more positive than prevailing beliefs and attitudes held by correctional staff; and (4) a "voluntary" community-based after-care treatment  *Community based after-care description not clear* | **Length**: Pre-release: 6-24 months; Post-release: 6-months  **Intensity**: Pre-release: 20 hours per week; Post-release: NR | **SAP**: those who participated in SAP but not in community after-care  **No treatment**: those who did not participate in SAP (no treatment group) | **Primary**: return-to-custody | 0.55 |
| Needels, 2005 | US, 1997-2000 | RCT | Jail and community | Adult females held in jails in New York City who showed a commitment to receiving services, were expected to be released to the community within 1 year, and were able to receive services in the South Bronx or Harlem, NY areas  (34.7) | N=1416 (49.7%, n=704)  JC n=352  J-only n=352 | **HealthLink – Jail-and-Community-Services (JC)**: A tailored set of services that would help address each client’s problems, called a discharge plan, became the guide for Health Link services after release. In addition, during frequent meetings held shortly after release from jail and then less frequently throughout the subsequent year, caseworkers supported clients by making referrals to services that could help clients address their substance abuse, health, and other problems; conducting crisis intervention; and providing counselling. | **Length**: Pre-release: up to 12-months; Post-release: 12-months  **Intensity**: Pre-release: NR; Post-release: voluntary (no set amount of sessions) | **Health Link – Jail-Services-Only (J-only)**: J group members were offered less-intensive discharge planning services and were ineligible for Health Link’s community case management services (but were encouraged to obtain services from other sources). | **Primary**: Health Link participation  **Secondary**: Criminal Justice System Involvement, Drug Treatment and Drug Use, Behaviours Affecting HIV Risk | 0.55 |
| Schram, 2002 | US, NR | Quasi-experimental design | Two correctional facilities and one prison camp, and in the community | Women were scheduled for parole in either Wayne or Oakland County, and had a prisoner classification that allowed them to live in the “general population”. Treatment group had at least six to nine months remaining prior to parole eligibility. Comparison group had <3 months remaining prior to eligibility for release  (Median range 31-40) | N=217 (100% female)  Treatment group n=146; Comparison group n=71 | **The Life Skills Program**: consists of five phases – 1. Eligible females are identified; 2. Assessment and program planning for each participant; 3. Implementation of the Life Skills Program: a transition counsellor hosts sessions that addresses: problem solving, stress and anger management, money and time management, self-esteem, negotiation skills, parenting, and employability skills; Phase 4 and 5 focused on reintegrating the participant back into the community. During Phase 4, an aftercare team develop a reintegration plan to identify the participant’s individual needs upon release; Phase 5 was the beginning of the participant’s release into the community. The first sixty days of release, the aftercare agency provides support and advocacy by an individual community advocate and limited financial assistance needed for housing and child care. | **Length**: Pre-release: 6-months; Post-release: 60-days  **Intensity**: Pre-release: three one-hour group sessions at least three days/week; Post-release: NR | **Comparison group**: Not clear | **Primary**: Group Differences in Life Skills  **Secondary**: Recidivism | 0.52 |

Note: CSC - Correctional Service of Canada; MMT – methadone maintenance treatment; PO – parole/probation officer; Post-release: intervention was post-release from jail/prison only; NA – not applicable; NR – not reported; NY – New York state; Post-release – community based; Pre-release – in-prison; RCT – randomised control trial; RTP – return to prison; SUD – substance-use disorder; TC – therapeutic community; Transitional – intervention was both pre- and post-release from jail/prison; US – United States
* 74% of the women participating in this investigation were on probation at some time during the 3 years post-release. Most of these women were enrolled in a specialized probation supervision program known as Promotion of Women through Education and Resources. POWER helps women change their lives by referring them to community-based services that address their addiction- and trauma-related needs, motivating and preparing them to continue their participation in intensive mental health and substance abuse treatments.
** the Correctional Service of Canada (CSC) is the federal government agency responsible for administering prison sentences of a term of two years or more, as imposed by the courts.
*** Data are not available for all the women who completed the WIT program
